# Inflammatory perturbations in early life long-lastingly shape the transcriptome and TCR repertoire of the first wave of regulatory T cells

**DOI:** 10.3389/fimmu.2022.991671

**Published:** 2022-08-31

**Authors:** Juhao Yang, Mangge Zou, Xiaojing Chu, Stefan Floess, Yang Li, Michael Delacher, Jochen Huehn

**Affiliations:** ^1^ Department Experimental Immunology, Helmholtz Centre for Infection Research, Braunschweig, Germany; ^2^ Roche Pharma Research and Early Development, Pharmaceutical Sciences, China Innovation Center of Roche, Shanghai, China; ^3^ Department Computational Biology for Individualised Medicine, Centre for Individualised Infection Medicine (CiiM), Helmholtz Centre for Infection Research and Hannover Medical School, Hannover, Germany; ^4^ Institute of Immunology, University Medical Center Mainz, Mainz, Germany; ^5^ Research Centre for Immunotherapy, University Medical Center Mainz, Mainz, Germany; ^6^ Cluster of Excellence RESIST (EXC 2155), Hannover Medical School, Hannover, Germany

**Keywords:** neonatal perturbations, regulatory T cells (Tregs), TCR repertoire, single-cell ‘omics, long-lasting

## Abstract

The first wave of Foxp3^+^ regulatory T cells (Tregs) generated in neonates is critical for the life-long prevention of autoimmunity. Although it is widely accepted that neonates are highly susceptible to infections, the impact of neonatal infections on this first wave of Tregs is completely unknown. Here, we challenged newborn Treg fate-mapping mice (Foxp3^eGFPCreERT2^xROSA26^STOP-eYFP^) with the Toll-like receptor (TLR) agonists LPS and poly I:C to mimic inflammatory perturbations upon neonatal bacterial or viral infections, respectively, and subsequently administrated tamoxifen during the first 8 days of life to selectively label the first wave of Tregs. Neonatally-tagged Tregs preferentially accumulated in non-lymphoid tissues (NLTs) when compared to secondary lymphoid organs (SLOs) irrespective of the treatment. One week post challenge, no differences in the frequency and phenotypes of neonatally-tagged Tregs were observed between challenged mice and untreated controls. However, upon aging, a decreased frequency of neonatally-tagged Tregs in both NLTs and SLOs was detected in challenged mice when compared to untreated controls. This decrease became significant 12 weeks post challenge, with no signs of altered Foxp3 stability. Remarkably, this late decrease in the frequency of neonatally-tagged Tregs only occurred when newborns were challenged, as treating 8-days-old mice with TLR agonists did not result in long-lasting alterations of the first wave of Tregs. Combined single-cell T cell receptor (TCR)-seq and RNA-seq revealed that neonatal inflammatory perturbations drastically diminished TCR diversity and long-lastingly altered the transcriptome of neonatally-tagged Tregs, exemplified by lower expression of *Tigit*, *Foxp3*, and *Il2ra*. Together, our data demonstrate that a single, transient encounter with a pathogen in early life can have long-lasting consequences for the first wave of Tregs, which might affect immunological tolerance, prevention of autoimmunity, and other non-canonical functions of tissue-resident Tregs in adulthood.

## Introduction

Accumulating evidence suggests that early-life infections can substantially shape the developing immune system (1). We have recently reported that acute *Listeria monocytogenes* infection in neonates causes long-term, organ-specific alterations of both the adaptive and innate immune system (2). Besides, childhood infections correlating with a beneficial long-lasting immune homeostasis include Epstein-Barr virus (EBV) and *Helicobacter pylori* infections, limiting the incidence of multiple sclerosis or asthma and allergy (3, 4). On the other hand, some infections can also promote the development of autoimmune diseases like type 1 diabetes, which has been linked to certain enteroviruses (5). In the same line, neonatal infections with respiratory syncytial virus, *Streptococcus pneumonia*, or Roseolovirus were shown to significantly increase allergic airway diseases during adulthood (6–8).

Regulatory T cells (Tregs) are defined as a suppressive CD4^+^ T cell subpopulation expressing the lineage-specifying transcription factor Foxp3. It is well-known that Foxp3^+^ Tregs are critical for preserving immune tolerance and preventing immunopathology, and altered Treg composition and/or dysfunction can lead to severe chronic infections, oncogenesis, and autoimmune diseases (9). Apart from Tregs in secondary lymphoid organs (SLOs), highly specialized Tregs in non-lymphoid tissues (NLTs), such as skin, liver, lung and adipose tissue, were more recently reported and are currently intensively investigated (10). These Tregs are termed tissue-resident Tregs and are characterized as ST2^+^KLRG1^+^ cells (11). Interestingly, tissue-resident Tregs harbor non-canonical functions such as tissue homeostasis and repair, suggesting that Tregs are not only regulatory as their name implies (12–17).

For the establishment of a fully functional Treg compartment, the neonatal period has been identified as a crucial time window (18). Particularly, an encounter with self-antigens and commensals during this period is essential to establish and maintain life-long immune homeostasis (19–21). In addition, it was demonstrated that neonatal CD4^+^ T cells have an intrinsic “default” mechanism to become Tregs in response to TCR stimulation (22) and that a temporal switch in negative selection and ligand binding kinetics constrains the neonatal tTreg selection window (23). Importantly, a recent study showed that neonatally-generated Tregs are indispensable for the life-long prevention of autoimmune diseases (24). The seeding of tissue-resident Tregs starts during the neonatal period and the selective blockage of this early-seeding process leads to tissue inflammation and abrogation of tolerance, indicative of a vital role of the first wave of tissue-resident Tregs in maintaining tissue homeostasis (20, 25). Recent studies revealed that the generation of tissue Tregs is a stepwise, multi-site process (26). It is initiated in SLOs, and the priming permits tissue Treg precursors to exit these sites and surveil NLTs, where a final specialization process takes place in response to unique microenvironmental cues (27–29). Despite this knowledge on the first wave of tissue-resident Tregs, the impact of neonatal infections on this highly relevant Treg compartment is unknown.

To fill this knowledge gap, we challenged newborn Foxp3^eGFPCreERT2^xROSA26^STOP-eYFP^ mice with Toll-like receptor (TLR) agonists to mimic inflammatory perturbations upon neonatal bacterial or viral infections, and selectively labeled the first wave of Tregs by repetitive administration of tamoxifen within the first 8 days of life. Interestingly, we observed a preferential accumulation of neonatally-tagged Tregs in NLTs when compared to SLOs regardless of the challenge with TLR agonists. Immediately after challenge (1 week), neonatally-tagged Tregs displayed comparable phenotypes with their counterparts from non-challenged control mice. However, upon aging a decreased frequency of neonatally-tagged Tregs emerged in both SLOs and NLTs of challenged mice, and became significant 12 weeks post challenge. Remarkably, this late impact on the first wave of Tregs in NLTs was not due to a loss of Foxp3 stability and was specific to inflammatory perturbations occurring in neonates. Combined single-cell (sc) RNA-seq and T cell receptor (TCR)-seq revealed that neonatal inflammatory perturbations persistently altered the transcriptome of neonatally-tagged Tregs and markedly diminished their TCR diversity. Together, our data demonstrate that neonatally-tagged Tregs preferentially accumulate in NLTs upon aging and that neonatal inflammatory perturbations have a negative and persistent impact on them, suggesting that a single encounter of pathogens in early life might have life-long consequences for immunological tolerance, prevention of autoimmunity and other non-canonical functions of tissue-resident Tregs.

## Materials and methods

### Mice

Foxp3^eGFPCreERT2^xROSA26^STOP-eYFP^ mice were bred and kept under specific pathogen-free conditions in isolated ventilated cages at the Helmholtz Centre for Infection Research (Braunschweig, Germany). Water and food were supplied ad libitum. In all experiments, gender- and age-matched mice were used. All mice were housed and handled in accordance with qualified animal practice as defined by FELASA and the national animal welfare body GV-SOLAS. All animal experiments were designed following the 3R principle and performed in accordance with the German Animal Welfare Act (TierSchG, TierSchVersV) and the European Union Directive 2010/63/EU, and were approved by the Lower Saxony Committee on the Ethics of Animal Experiments as well as the state office (Lower Saxony State Office of Consumer Protection and Food Safety) under the permit number 33.19-42502-04-17/2382.

### Neonatal inflammatory perturbations and neonatal tagging of Tregs

Newborn mice (less than 24-hours-old) were carefully fixed by hand and intraperitoneally injected with 2 µg LPS (List labs) or 20 µg poly I:C (Sigma) per gram mouse in 20 µl PBS. To label the first wave of Tregs, 100 µg tamoxifen (Sigma) dissolved in 50 µl peanut oil (Sigma) was intragastrically administrated to mice on days 2, 5, and 8 after birth.

### Flow cytometry

Flow cytometric analysis was performed as described recently (30). In brief, single-cell suspensions were washed with PBS before dead cells were labeled with the UV-excitable, fixable Live/Dead dye (Thermo Fisher) for 30 min staining at 4°C. Surface staining was performed for 15 min on ice in PBS (Gibco) containing 0.2% bovine serum albumin (BSA, Sigma-Aldrich) and 5 mM EDTA (Roche). For transcription factor staining, cells were subsequently fixed with 4% paraformaldehyde (Merck) at room temperature for 30 min in the dark, permeabilized using eBioscience™ Permeabilization Buffer (Thermo Fisher) according to the manufacturer’s protocol, and stained intracellularly for 30 min or overnight at 4°C. All flow cytometry samples were acquired on a LSR Fortessa (BD Biosciences), and data were analyzed using FlowJo^®^ 9.9.6 (FlowJo, LLC).

### Lymphocytes isolation

To isolate cells from the spleen and LNs, organs were disintegrated through a 30 µM cell sieve (Sysmex Partec), and splenocytes were further cleared from erythrocytes by lysis. Cells were resuspended in PBS (Gibco) containing 0.2% BSA and 5 mM EDTA and were kept on ice until further processing.

To isolate cells from liver or lung tissues, animals were perfused by opening the inferior vena cava and flushing the left ventricle with PBS to get rid of blood from the circulation. Next, tissues were collected and cut into small pieces, which were digested in HBSS medium (Gibco) containing 25 mM HEPES (Biochrom), 1 mg/ml collagenase D (Roche), and 0.1 mg/ml DNase I (Roche) for 60 min at 37°C. To purify lymphocytes, cells were resuspended in 40% Percoll (GE Healthcare) and layered on top of an 80% Percoll layer. The mixture was centrifuged at 780×g at 20°C for 20 min without brake. Finally, lymphocytes were collected from the interface between the two layers.

To isolate cells from colon tissues, colons were opened longitudinally and washed with PBS to remove feces. Epithelial cells and intraepithelial lymphocytes were removed by washing with pre-digestion buffer (PBS containing 0.2% w/v BSA and 5 mM EDTA) for 30 min at 37°C. After extensive washing with PBS to get rid of the remaining EDTA, colons were cut into small pieces and digested in HBSS medium containing 25 mM HEPES, 1 mg/ml collagenase D, and 0.1 mg/ml DNase I for 60 min at 37°C. Lamina propria lymphocytes were purified with a 40%/80% Percoll gradient as described above.

To isolate cells from skin tissues, hair and hair follicles from the back of the animal were removed with an electric shaver and depilatory cream (Veet). The skin was separated from the dorsal surface, cut into small pieces, and digested in HBSS medium containing 25 mM HEPES, 1 mg/ml collagenase D, and 0.1 mg/ml DNase I in a GentleMACS C tube (Miltenyi Biotec) and the program ‘‘37_C_Multi_H’’ for 90 min, followed by centrifugation and the abovedescribed 40%/80% Percoll gradient for the enrichment of lymphocytes.

### scRNA-seq and scTCR-seq

Single-cell suspensions were prepared from spleen, colon, and skin of 12-weeks-old mice that had been neonatally treated with LPS, poly I:C or PBS as control. With the exception of splenocytes, cells were further stained with TotalSeq C hashtag antibodies (BioLegend) according to the manufacturer’s protocol. The same type of organs was labeled by the same TotalSeq Hashtag antibodies, and afterwards spleen, colon, and skin samples from the same mouse were pooled for sorting CD8^-^CD45R^-^CD11c^-^CD11b^-^NK1.1^-^CD4^+^YFP^+^ cells. Subsequently, sorted cells were loaded onto a 10x Chromium Next GEM Chip G using reagents from the Chromium Single Cell 5’ Library and Gel Bead Kit (10x Genomics) according to the manufacturer’s protocol. Amplified cDNA was used for both 5’ RNA-seq library generation and TCR V(D)J targeted enrichment using the Chromium Single-Cell V(D)J Enrichment Kit for Mouse T Cells (10x Genomics). 5’ RNA-seq and TCR V(D)J libraries were prepared following the manufacturer’s user guide (10x Genomics). Libraries were quantified by QubitTM 3.0 Fluorometer (ThermoFisher) and quality checked using 2100 Bioanalyzer with High Sensitivity DNA Kit (Agilent Technologies). scRNA-seq libraries were sequenced on an Illumina NextSeq500 sequencer to attain approximately 141,381 ± 63,000 reads per single cell and scTCR V(D)J libraries were sequenced on an Illumina NextSeq500 sequencer to obtain approximately 63,987 ± 6,131 reads per cell. The sequencing specifications for both RNA and TCR V(D)J libraries were according to the manufacturer’s specification (10x Genomics).

### scRNA-seq analysis

Cell Ranger (v.3.0.2) was applied to align reads (mouse GRCm38/mm10 as reference genome) and to produce gene-barcode matrices for each sample. Quality control and cell clustering were performed using Seurat (version 3.1.2) (31). To filter out doublets and cells with low quality, cells expressing genes more than 2,000, with more than 5,000 counts detected, or mitochondrial genes with more than 5% of total unique molecular identifiers (UMIs) were removed from the downstream analysis. Next, gene expression levels were normalized *via* the NormalizeData function, and the top 2,000 variable genes were identified using the FindVariableFeatures function. To reduce dimensions, principal component analysis (PCA) was applied to scale data with PC 1-15 and resolution 0.5. Notably, given that unhashtaged cells contain cells of the spleen and cells from other tissues that failed in hashtagging, we further applied empirical filtering criteria and excluded unhashtaged cells clustered with other tissues leaving the majority of unhashtag cells clustered separately from other tissues for the further analysis. We next combined samples based on their batches and filtered out contaminating cells, characterized by the expression of non-T cell makers including *Cd19*, *Ncr1*, *Adgre1*, *Cd14*, *Itgax*, *Ly6g*, *Pdpn*, *Ebf1*, *Ebf2*, *Ebf3*, *Iglc3*, *Iglc2*, *Iglc1*, *Iglc4*, *Igkv17-127*, *Cd79b*, *Cd79a*, *Jchain*, *Lyz2*, *Igkv10-94*, *Fcer1g*, *Tyrobp*, *Ccl6*, *Slpi*, *Trdc*, *Gpr25*, *Cxcl10*, *Bst2*, *H2-Eb1*, *H2-Eb2*, *H2-Ab1*, *H2-Aa*, *Hbb-bs*, *Gm30211*, *Igkc*, and *Apoe*. Afterwards, we re-normalized the data and identified the top 2,000 variable genes. Subsequently, we performed the unsupervised clustering with the same settings (PC1-15, resolution = 0.5). Furthermore, we zoomed into the data of each tissue and identified subclusters using the same PC numbers and resolution. Cell clusters were visualized using Uniform Manifold Approximation and Projection (UMAP).

To identify differentially expressed genes (DEGs) for each treatment group and by taking samples from PBS-treated mice as control, the FindMarkers function was applied with the default Wilcoxon rank sum test and P-value adjustment performed using Bonferroni correction based on the total number of genes in the tested dataset. Genes were considered differentially expressed when the logFC > 0.25, expressed in >10% of cells in the cluster, and the adjusted P-value < 0.05.

### scTCR-seq analysis

The Cell Ranger vdj pipeline was applied to assemble the TCR sequences and identify the CDR3 sequence and TCR genes. Taking the clean scRNA-seq data, we further removed cells that failed in detecting both α and β chains for further analysis. A clonal size was identified by the number of cells expressing the same α and β chains.

### Statistical analysis

Prism software (GraphPad) was utilized for the statistical analysis of flow cytometry data. For all figures, if not stated otherwise, each data point represents a single mouse. When unmatched groups were compared, a One-way ANOVA Kruskal-Walis test was applied. Data are presented as mean or mean ± SD, and p-values below a threshold of 0.05 were considered as significant (* p < 0.05; ** p < 0.01; *** p < 0.001; **** p < 0.0001; ns = not significant).

## Results

### Neonatally-tagged Tregs accumulate with distinct kinetics in SLOs and NLTs

**Figure 1:**
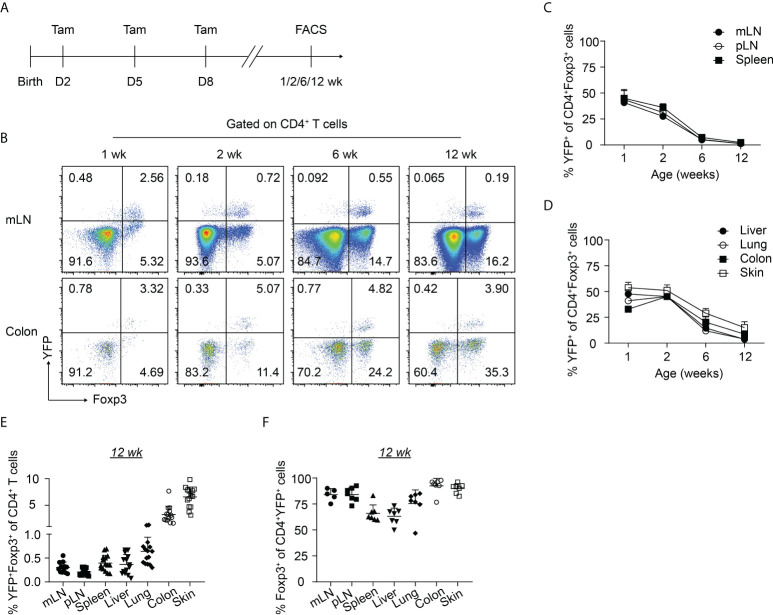
Kinetics of neonatally-tagged Tregs in different organs. Foxp3^eGFPCreERT2^xROSA26^STOP-eYFP^ mice received repetitive intragastric tamoxifen injections at days 2, 5 and 8 after birth, and were sacrificed at indicated time points (1, 2, 6, and 12 weeks corresponding to days 8, 15, 43, and 85 after birth, respectively). Single-cell suspensions from mLN, pLN, spleen, colon, skin, liver, and lung were analyzed by flow cytometry. **(A)** Scheme of experimental design. **(B)** Exemplary pseudocolor plots display Foxp3 and YFP expression among CD4^+^ T cells isolated from mLN (top) and colonic lamina propria (bottom) at indicated time points. **(C, D)** Kinetic accumulation of neonatally-tagged YFP^+^ Tregs in mLN, pLN, and spleen **(C)**, and in colon, skin, liver, and lung **(D)**. **(E)** Scatter dot plot shows frequencies of YFP^+^Foxp3^+^ cells among CD4^+^ T cells in indicated organs of 12-weeks-old mice. **(F)** Scatter dot plot shows frequencies of Foxp3^+^ cells among CD4^+^YFP^+^ T cells in indicated organs of 12-weeks-old mice. Data in scatter dot plots are depicted as mean ± SD, each dot represents a single mouse, and data were pooled from 3 to 6 independent experiments (n = 6-8).

To gain insight into the dynamics of the neonatally-generated Treg compartment, we employed Treg fate-mapping mice (Foxp3^eGFPCreERT2^xROSA26^STOP-eYFP^) (32). In these mice, the ubiquitously expressed *ROSA26* locus contains a loxP site-flanked STOP cassette followed by a DNA sequence encoding yellow fluorescent protein (YFP), and the eGFP-CreERT2 fusion protein is sequestered in the cytosol, wherefore YFP is not expressed. Upon tamoxifen treatment, eGFP-CreERT2 is translocated into the nucleus, allowing the excision of the floxed STOP cassette and subsequently resulting in the constitutive and heritable expression of YFP in a cohort of cells that expressed Foxp3 at the time of tamoxifen administration (32). Using these Treg fate-mapping mice and applying a published protocol for neonatal tamoxifen administration (33), we selectively labeled the first wave of Tregs in neonatal mice by repetitive intragastric tamoxifen injections on days 2, 5, and 8 after birth. Subsequently, we determined the distribution and immunophenotype of the neonatally-tagged Tregs in SLOs and NLTs at 1, 2, 6 and 12 weeks after birth by flow cytometry (**Figure 1A**, **Supplementary Figure 1**). Consistent with previously published data (24), YFP labeling efficiency in neonatal mice (1 week) is about 40-50% in mLN and 30% in the colon (**Figures 1B, C**). As expected, the pool of neonatally-tagged Tregs in SLOs was rapidly diluted upon aging by newly generated, non-labeled Tregs, reflected by a gradual drop of the percentage of YFP^+^ Tregs from 40-50% at the 1-week time point to less than 1% after 12 weeks (**Figures 1B, C**). However, neonatally-tagged Tregs showed distinct kinetics in NLTs. At the 1-week time point, the percentage of YFP^+^ Tregs was highest in the skin with more than 50% YFP^+^ cells among total Tregs, followed by liver, lung, and colon (**Figure 1D**). In contrast to SLOs, the pool of neonatally-tagged Tregs in NLTs was not rapidly diluted upon aging by newly generated, non-labeled Tregs, but rather showed a constant or even higher frequency of YFP^+^ Tregs in 2-weeks- when compared to 1-week-old mice (**Figure 1D**), indicating a further enrichment or migration of neonatally-generated Tregs into NLTs. Although the dilution effect also became apparent in NLTs at later time points (6 and 12 weeks), a generally higher frequency of YFP^+^ Tregs was observed at these sites when compared to SLOs (**Figures 1D, E**). This difference was not due to a conversion of Foxp3^+^ Tregs into exFoxp3^+^ cells due to a loss of Foxp3 expression since a comparable frequency of Foxp3^+^ cells among YFP^+^CD4^+^ T cells was observed in SLOs and NLTs at 12 weeks after birth, except for a lower Foxp3 stability among YFP^+^ Tregs in liver and spleen (**Figure 1F**). Together, our data demonstrated the distinct kinetics of neonatally-tagged Tregs between SLOs and NLTs.

### Neonatally-tagged Tregs display higher frequencies of activated, tissue-resident cells compared to adult Tregs

**Figure 2:**
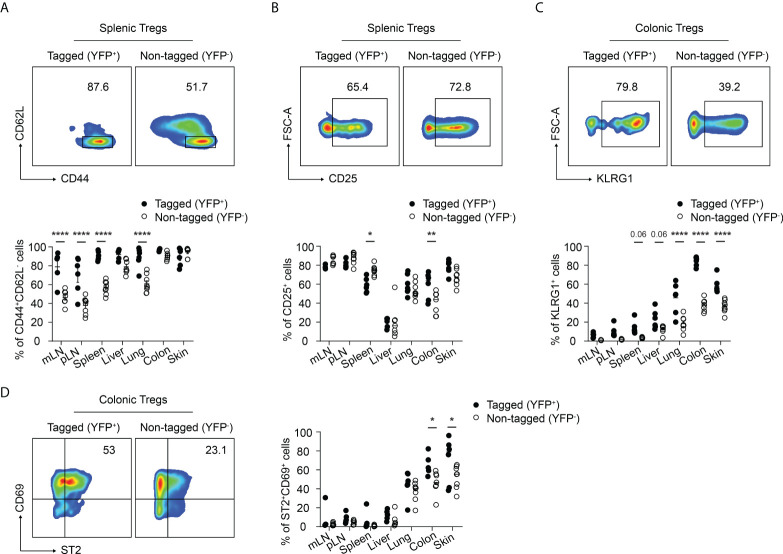
Immunophenotype of neonatally-tagged Tregs in adult mice. Foxp3^eGFPCreERT2^xROSA26^STOP-eYFP^ mice received repetitive intragastric tamoxifen injections at days 2, 5 and 8 after birth, and were sacrificed for immunophenotyping at 12 weeks (day 85) after birth. **(A–D)** Representative plots show CD62L and CD44 expression **(A)**, CD25 expression **(B)**, KLRG1 expression **(C)**, and CD69 and ST2 expression **(D)** among non-tagged (YFP^-^) and tagged (YFP^+^) Foxp3^+^ Tregs from indicated organs. Scatter dot plots summarize the frequencies among non-tagged (open circle) and tagged Tregs (black circle) across indicated organs. Data in scatter dot plots are depicted as mean ± SD, each dot represents a single mouse, and data were pooled from 3 to 6 independent experiments (n = 6-8). * p < 0.05, ** p < 0.01, **** p < 0.0001.

Next, we further characterized the phenotype of the neonatally-tagged Tregs in SLOs and NLTs of 12-weeks-old mice. Neonatally-tagged Tregs expressed higher CD44 levels and displayed a more pronounced effector/memory phenotype when compared to non-labeled YFP^-^ Tregs (**Figure 2A**). The high activating state indicates that these neonatally-tagged Tregs continuously received stimulating signals *in vivo* to maintain their survival, wherefore we further checked for the expression of CD25, the high-affinity IL 2 receptor. As reported before (25), liver-residing Tregs exhibited rather low CD25 expression levels in both neonatally-tagged and non-labeled Tregs (**Figure 2B**). On the contrary, Tregs of pLN, mLN, and skin displayed the highest CD25 expression, with no difference detected between YFP^+^ and YFP^-^ Tregs. Interestingly, differences in CD25 expression were observed in the spleen and colon, with YFP^+^ Tregs showed a lower and higher expression, respectively, when compared to YFP^-^ Tregs (**Figure 2B**). The preferential accumulation of neonatally-tagged Tregs in NLTs prompted us to assess the expression of tissue residency markers. As expected (10, 11), increased frequencies of KLRG1^+^ and ST2^+^CD69^+^ cells were found among Tregs from NLTs, particularly lung, colon and skin, when compared to Tregs from SLOs. Interestingly, significantly larger fractions of KLRG1^+^ and ST2^+^CD69^+^ cells were observed among neonatally-tagged Tregs when compared to non-labeled Tregs (**Figures 2C, D**), suggesting the higher frequency of YFP^+^ Tregs in NLTs being a consequence of the retention of these cells in the tissues.

### Neonatal inflammatory perturbations reduce the abundance of neonatally-tagged Tregs in SLOs and NLTs during adulthood

**Figure 3:**
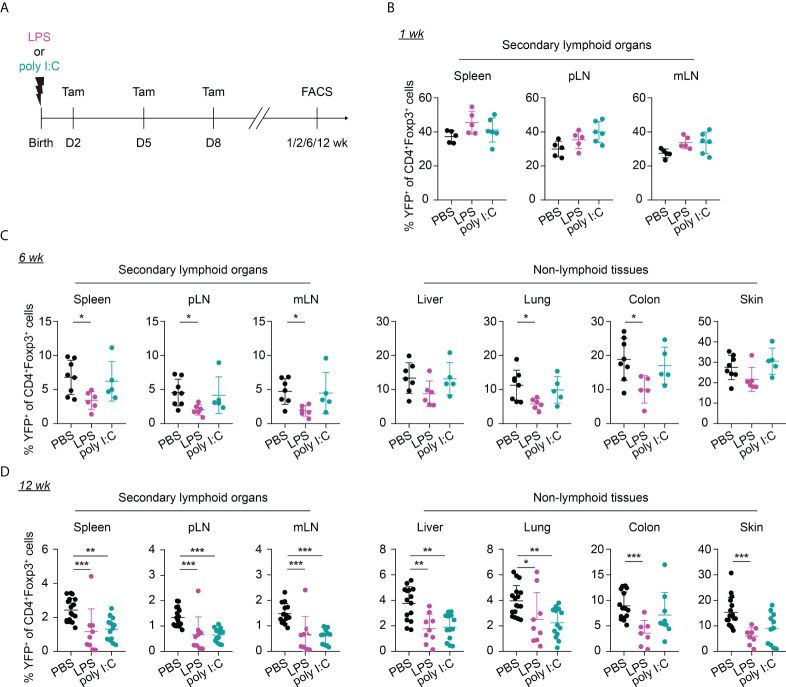
Long-term impact of neonatal inflammatory perturbations on neonatally-tagged Tregs. **(A)** Newborn Foxp3^eGFPCreERT2^xROSA26^STOP-eYFP^ mice were intraperitoneally injected with LPS, poly I:C or PBS as control, received repetitive intragastric injections of tamoxifen at days 2, 5 and 8 after birth, and were sacrificed at indicated time points after challenge. **(B–D)** Scatter dot plots summarize frequencies of neonatally-tagged (YFP^+^) Tregs among Foxp3^+^ Tregs across indicated organs of LPS- (purple), poly I:C- (turquoise) or PBS-treated mice (black) 1 **(B)**, 6 **(C)** and 12 **(D)** weeks post inflammatory perturbation. Data in scatter dot plots are depicted as mean ± SD, each dot represents a single mouse, and data were pooled from 4 to 6 independent experiments (n = 5-11). * p < 0.05, ** p < 0.01, *** p < 0.001.

Given that neonates are extremely susceptible to infections, we sought to investigate the potential impact of neonatal infections on the first wave of Tregs and their phenotype during aging. To this end, we first established neonatal challenge models by intraperitoneal administration of LPS or poly I:C into newborns to mimic inflammatory perturbations upon neonatal acute bacterial or viral infections, respectively (**Figure 3A**). Dose titration studies revealed that neonates either succumbed within the first 2 days after treatment or rapidly recovered (**Supplementary Figure 2**), confirming the suitability of these inflammatory perturbation models.

**Figure 4:**
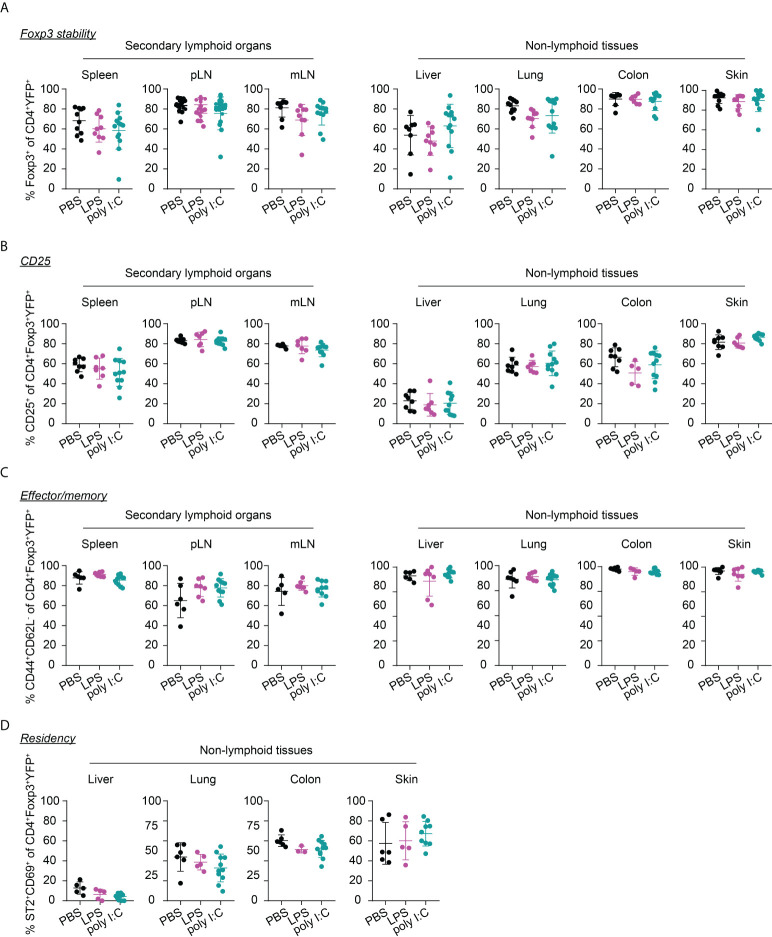
Immunophenotype of neonatally-tagged Tregs 12 weeks post neonatal inflammatory perturbation. Newborn Foxp3^eGFPCreERT2^xROSA26^STOP-eYFP^ mice were intraperitoneally injected with LPS, poly I:C or PBS as control, received repetitive intragastric injections of tamoxifen at days 2, 5 and 8 after birth, and were sacrificed at indicated time points after challenge. Scatter dot plots depict frequencies of Foxp3^+^ cells among CD4^+^YFP^+^ T cells **(A)**, and of CD25^+^ cells **(B)**, CD44^+^CD62L^-^ cells **(C)**, and ST2^+^CD69^+^ cells **(D)** among neonatally-tagged YFP^+^Foxp3^+^ Tregs across indicated organs. Data in scatter dot plots are depicted as mean ± SD, each dot represents a single mouse, and data were pooled from 4 to 6 independent experiments (n = 6-11).

Interestingly, the neonatal inflammatory perturbations had no immediate impact on the first wave of Tregs since a comparable frequency of YFP^+^ cells among Tregs was observed at the 1-week time point in challenged mice when compared to untreated controls (**Figure 3B**). However, the neonatal inflammatory perturbations showed unexpected late effects as LPS-treated mice started to show significantly lower frequencies of YFP^+^ cells among Tregs in SLOs and at least a trend in NLTs at the 6-weeks time point (**Figure 3C**). These perturbation-driven effects were further amplified upon aging, and both LPS- and poly I:C-treated mice showed significantly lower frequencies of neonatally-tagged Tregs in SLOs and NLTs at 12 weeks post inflammatory perturbation (**Figure 3D**). Again, we could exclude the possibility that the reduced fraction of YFP^+^ cells is due to a conversion of Foxp3^+^ Tregs into exFoxp3^+^ cells due to a loss of Foxp3 expression since both the control and challenged groups showed comparable Foxp3 stability and CD25 expression in SLOs and NLTs (**Figures 4A, B**). Additionally, transient neonatal inflammatory perturbations barely generated any long-term impact on the effector/memory or tissue residency phenotype of neonatally-tagged Tregs at 12 weeks post inflammatory perturbation (**Figures 4C, D**). Taking together, acute neonatal inflammatory perturbations affect the abundance of neonatally-generated Tregs in both SLOs and NLTs during adulthood without gross effects on their effector/memory and tissue residency phenotype.

### Slightly delayed inflammatory perturbations less strongly affect the abundance of neonatally-tagged Tregs in SLOs and NLTs during adulthood

**Figure 5:**
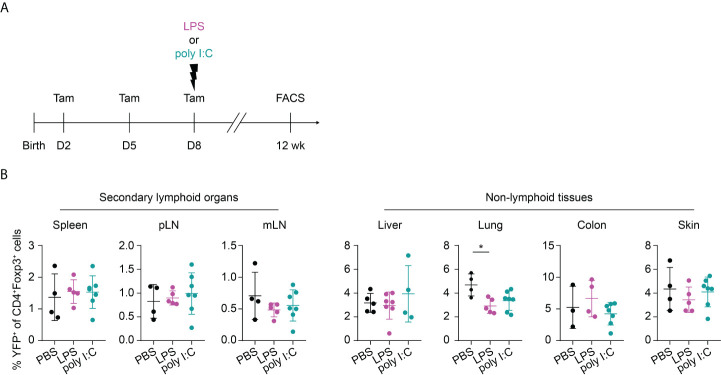
No long-term impact on neonatally generated Tregs upon later challenge. **(A)** Foxp3^eGFPCreERT2^xROSA26^STOP-eYFP^ mice received repetitive intragastric tamoxifen injections at days 2, 5 and 8 after birth, directly followed by an intraperitoneal injection of LPS, poly I:C or PBS on day 8. Twelve weeks later, mice were sacrificed for immunophenotyping. **(B)** Scatter dot plots summarize frequencies of YFP^+^ cells among Foxp3^+^ Tregs in secondary lymphoid organs (left) and non-lymphoid tissues (right). Data in scatter dot plots are depicted as mean ± SD, each dot represents a single mouse, and data were pooled from 2 independent experiments (n = 4-7). * p < 0.05.

Recently, common precursors for tissue-resident Tregs were identified in SLOs with a peak at day 10 after birth before their migration into the peripheral tissues (27). We wondered whether a perturbation occurring shortly before the peak of the tissue Treg precurcors would even amplify the perturbation-induced, impaired residency of neonatally-tagged Tregs in NLTs. To this end, we shifted the inflammatory perturbation from day 1 to day 8 and determined the phenotype of neonatally-tagged Tregs when the mice have reached the age of 12 weeks (**Figure 5A**). Although the inflammatory perturbations occurred shortly before the peak of the tissue Treg precursors, both LPS- and poly I:C-treated groups unexpectedly showed an unaltered abundance of YFP^+^ cells among total Tregs in NLTs and SLOs when compared to the non-challenged controls (**Figure 5B**), and also the frequency of total Foxp3^+^ Tregs was not affected (**Supplementary Figure 3**). Together, our data suggest that the impaired abundance of neonatally generated Tregs in SLOs and NLTs during adulthood is restricted to inflammatory perturbations occurring immediately after birth and these negative effects are unlikely mediated through an impaired development of tissue Treg precursors.

### Neonatal inflammatory perturbations persistently alter the transcriptome of neonatally-tagged Tregs

**Figure 6:**
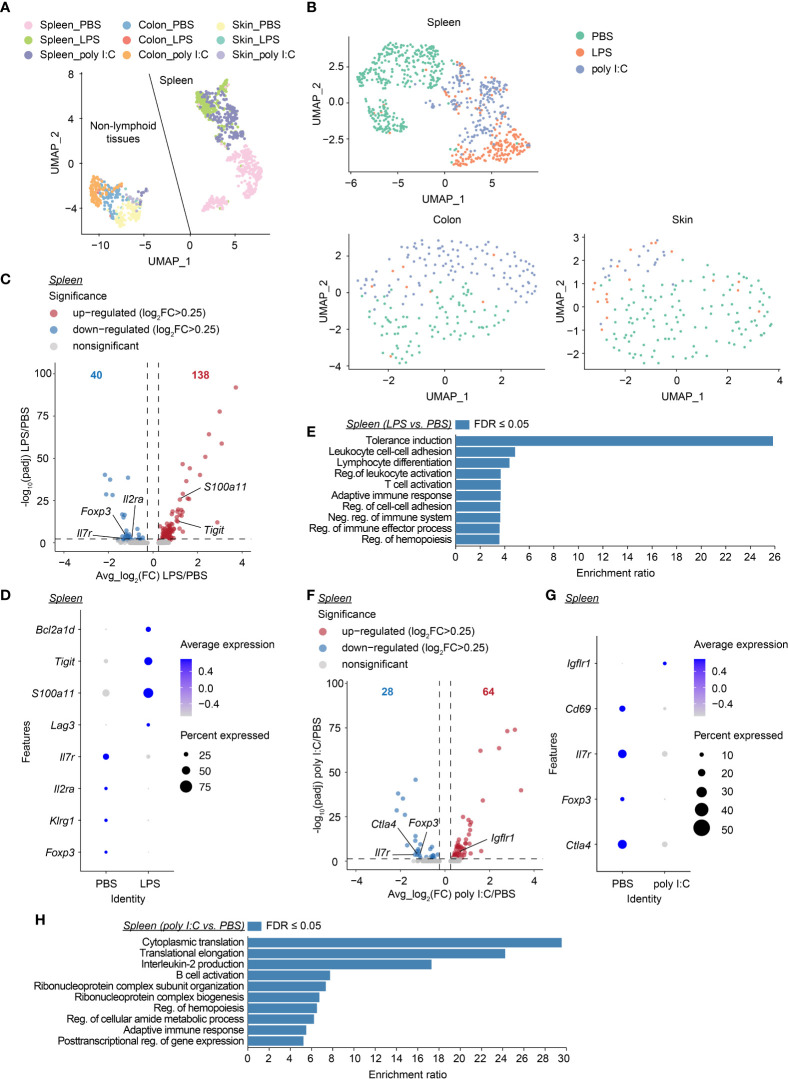
Long-lastingly altered transcriptomic profiles of neonatally-tagged Tregs upon neonatal challenge. Newborn Foxp3^eGFPCreERT2^xROSA26^STOP-eYFP^ mice were intraperitoneally injected with LPS, poly I:C or PBS as control, followed by repetitive intragastric injections of tamoxifen at days 2, 5 and 8 after birth. Twelve weeks later, YFP^+^ cells were FACS-sorted and subjected to combined scRNA/TCR-seq. **(A)** UMAP plot of merged YFP^+^ cells from spleen, colon, and skin of the first batch of PBS-, LPS- and poly I:C-treated mice. **(B)** UMAP plots of merged YFP^+^ cells across organs of PBS-, LPS- and poly I:C-treated mice. **(C)** Volcano plot depicts avg_log_2_(FC) *vs*. -log_10_(padj) of identified DEGs between LPS and PBS conditions in the spleen. **(D)** Dot plot shows the expression of selected genes in splenic YFP^+^ cells of PBS- and LPS-treated groups. **(E)** GO analysis of all DEGs identified in **(C, F)** Volcano plot depicts avg_log_2_(FC) *vs*. -log_10_(padj) of identified DEGs between poly I:C and PBS conditions in the spleen. **(G)** Dot plot shows the expression of selected genes in splenic YFP^+^ cells of PBS- and poly I:C-treated groups. **(H)** GO analysis of all DEGs identified in **(F)** UMAP, uniform manifold approximation, and projection; FC, fold change; DEG, differentially expressed gene; GO, gene ontology; Reg., regulation; Neg., negative.

Next, we aimed to further unravel the persistent effects of transient neonatal inflammatory perturbations on the first wave of Tregs at the molecular level. At the age of 12 weeks, neonatally-tagged YFP^+^ Tregs were sorted from the spleen, colon, and skin of neonatally LPS-, poly I:C- or PBS-treated mice, and in-depth transcriptional analysis was performed by scRNA-seq combined with scTCR-seq. Two independent datasets from distinct donors were generated for all treatment groups. We first used uniform manifold approximation and projection (UMAP) for dimensionality reduction to relate neonatally-tagged Tregs sorted from distinct organs. Interestingly, major segregation between spleen-sorted cells and cells isolated from colon and skin was observed, regardless of treatment (**Figure 6A**, **Supplementary Figure 4A**), indicative of a transcriptomic reprogramming of the first wave of Tregs in NLTs to facilitate their adaption and retention. Next, we determined the perturbation-driven effects on the transcriptome of neonatally-tagged Tregs across organs. Remarkably, both spleen and NLT samples showed distinct segregation between challenged mice and untreated controls (**Figure 6B**, **Supplementary Figure 4B**). Due to the limited cell number obtained from colon and skin, the further treatment-pairwise analysis only focused on the spleen samples. More than 150 genes showed differential expression as late as 12 weeks post neonatal LPS treatment, exemplified by *Tigit*, *Foxp3*, and *Il2ra* expression (**Figures 6C, D**, **Supplementary Figures 4C, D**). Gene ontology (GO) analysis of these genes revealed an enrichment of genes involved in ‘tolerance induction’ (**Figure 6E**), suggesting that neonatal LPS treatment might persistently affect the functionality of neonatally-generated Tregs. Similar phenotypes were observed in poly I:C-treated mice, with the expression of a bunch of genes including *Foxp3* being differentially expressed between challenged mice and untreated controls as late as 12 weeks post neonatal treatment (**Figures 6F, G**, **Supplementary Figures 4E, F**). GO analysis indicated an involvement of multiple biologic processes including ‘adaptive immune response’ (**Figure 6H**). Furthermore, we validated the lower expression of CD25 and Foxp3 at the protein level *via* flow cytometry (**Supplementary Figure 5**). Overall, transient neonatal inflammatory perturbations resulted in persistently altered transcriptional profiles of neonatally-generated Tregs.

### Neonatal inflammatory perturbations diminish the TCR diversity of neonatally-tagged Tregs

**Figure 7:**
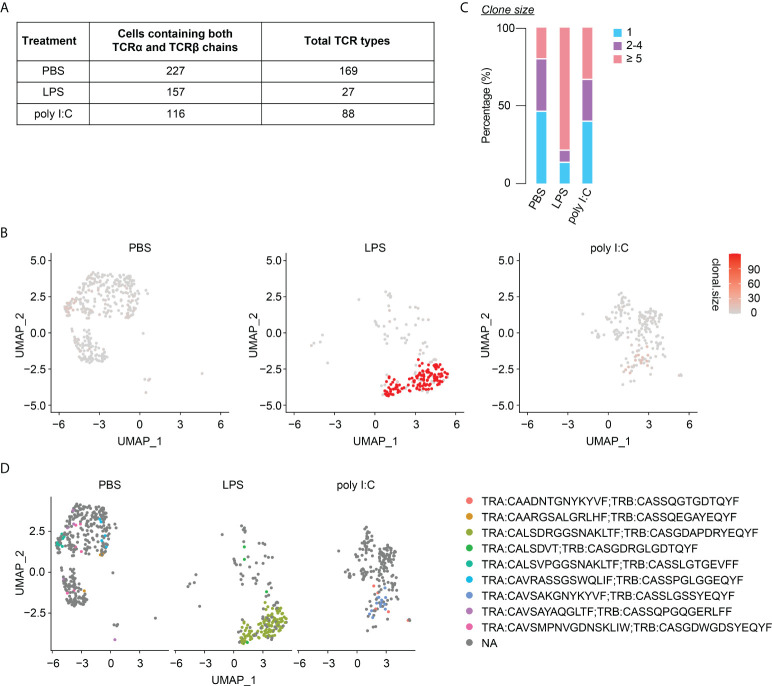
Diminished TCR diversity among neonatally-tagged Tregs 12 weeks post neonatal challenge. Newborn Foxp3^eGFPCreERT2^xROSA26^STOP-eYFP^ mice were intraperitoneally injected with LPS, poly I:C or PBS as control, followed by repetitive intragastric injections of tamoxifen at days 2, 5 and 8 after birth. Twelve weeks later, YFP^+^ cells were FACS-sorted and subjected to combined scRNA/TCR-seq. **(A)** Table of total TCR clonotypes detected among splenic YFP^+^ cells with paired TCRα and TCRβ chains detected in PBS-, LPS- and poly I:C-treated mice. **(B)** UMAP plots depict the clonal size in splenic YFP^+^ cells. **(C)** Bar plot summarizes frequencies of cells with unique (n = 1) or clonal TCR (n = 2-4 and n ≥ 5). **(D)** Splitted UMAPs depict clonotypes detected in clonal YFP^+^ cells (n ≥ 5). UMAP, uniform manifold approximation and projection.

Finally, we investigated the long-lasting consequences of neonatal inflammatory perturbations on the TCR diversity of neonatally-generated Tregs. Of the 227, 157 and 116 identified neonatally-tagged splenic Tregs from the first batch of PBS-, LPS- and poly I:C-treated mice, for which both TCRα and TCRβ chains could be detected, 169, 27, and 88 clonotypes were identified, respectively (**Figure 7A**). The substantially reduced TCR diversity in neonatally-tagged Tregs upon LPS treatment was also confirmed in the second batch of samples (**Supplementary Figure 6A**), whereas the effect of neonatal poly I:C treatment on the TCR diversity was less severe (**Figure 7A**, **Supplementary Figure 6A**). In line with a decreased TCR diversity upon LPS treatment, an enrichment of cells with clonal TCR expansion (n≥5) was observed in neonatally-tagged Tregs of LPS-treated mice (**Figures 7B, C**, **Supplementary Figures 6B, C**). Interestingly, the majority of these highly amplified cells were of the same TCR clonotype (**Figure 7D**, **Supplementary Figure 6D**). Taking together, neonatal inflammatory perturbations led to a decreased TCR diversity among neonatally-generated Tregs with the amplification of specific TCR clones.

## Discussion

Recently, the crucial role of the first wave of Tregs in preventing autoimmunity and in mediating tolerance towards commensals has been intensely studied (20, 24, 25). However, a detailed characterization of these neonatally-generated Tregs is still lacking. Within this study, we characterized the distribution and phenotype of the first wave of Tregs in both SLOs and NLTs during aging. Meanwhile, we investigated the impact of neonatal inflammatory perturbations on the phenotype of the first wave of Tregs and could demonstrate that acute neonatal inflammatory perturbations long-lastingly shifted the abundance of neonatally-tagged Tregs in SLOs and NLTs. Remarkably, these long-term effects were restricted to a very narrow early-life time window as inflammatory perturbations occurring on day 8 were already too late to instigate the same sequelae. Combined scRNA/TCR-seq further revealed that a single inflammatory perturbation in newborns not only persistently altered the transcriptome of neonatally-tagged Tregs, but also caused a strong reduction in their TCR diversity. Taking together, our data demonstrate long-lasting consequences of a single transient inflammatory perturbation during the neonatal period for the first wave of Tregs.

By restricting the tamoxifen administration to the first 8 days of life, we specifically labeled the first wave of Tregs and determined their kinetical distribution across organs. The population of neonatally-tagged Tregs could be stably detected in both SLOs and NLTs as late as 12 weeks after birth, demonstrating their remarkable persistence. As reported recently (34), ‘old’ Tregs exhibited an activated phenotype, implicating that continuous TCR signal supports their long life. Yet, also IL-2 signaling might contribute to their long living as neonatally-tagged Tregs display a universally high CD25 expression in almost all organs besides the liver. Subsequent to the withdrawal of tamoxifen treatment, the fraction of neonatally-tagged YFP^+^ Tregs among total Tregs gradually declined over time, most likely due to continuous thymic output of newly generated YFP^-^ Treg cells. Yet, during the first 2 weeks of life neonatally-tagged Tregs continuously accumulated in NLTs before being diluted in later life. This persistent accumulation of the first wave of Tregs in NLTs is in accordance with the physiological accumulation pattern of bulk Tregs in the skin and liver, for which a peak in the second week after birth was reported (20, 25).

The first wave of Tregs preferentially resides in NLTs. Additionally, we demonstrated that a single inflammatory perturbation in newborns is capable of not only impairing the residency of the first wave of Tregs in NLTs, but also negatively affecting the abundance of neonatally-tagged Tregs circulating through SLOs. Strikingly, this common decrease of the first wave of Tregs was not observed 1 week after challenge, but appeared during aging with mild effects after 6 weeks and significant differences 12 weeks after challenge. So far, the mechanisms underlying this long-lasting decrease of neonatally-tagged Tregs remain unclear. One possibility is that the tissue niches might be modulated by the early-life inflammatory perturbations and that Tregs are highly sensitive to these changes. Therefore, it is of interest to investigate whether the long-lasting effects mediated by neonatal inflammatory perturbations are directly acting on Tregs or indirectly *via* accessory, niche-forming cells.

Several studies have described unique tissue-specific adaptations of Treg, as reflected by distinct chromatin accessibility, epigenetic modification, and transcriptome in NLT-Tregs when compared to their counterparts from SLOs (11, 27, 35, 36). Here, tissue-specific adaptions were also observed in long-living, neonatally-tagged Tregs, best exemplified by their unique transcriptional signatures. Interestingly, a single transient inflammatory perturbation in neonates was sufficient to further modify this tissue-specific adaptation process by persistently altering the transcriptome of neonatally-tagged Tregs not only in NLTs, but also in SLOs. For instance, *Foxp3* and *Il2ra* expression were persistently down-regulated as a consequence of the neonatal inflammatory perturbation. Although downregulation of Foxp3 and high-affinity IL-2 receptor CD25 expression by Tregs had been reported before in various infection- or autoimmune diseases-associated inflammatory milieus (37–39), to the best of our knowledge we are the first to demonstrate that the inflammation-driven downregulation of *Foxp3* and *Il2ra* could persist far beyond the resolution of the inflammation. Foxp3 and CD25 are predominant regulators for Treg development, stability, function, and homeostasis (40–43), yet we could exclude Treg instability as a potential cause of lower *Foxp3* and *Il2ra* expression as both neonatally challenged and untreated mice showed comparable Foxp3 stability among neonatally-tagged Tregs. However, we wonder whether the downregulation of *Foxp3* and *Il2ra* expression would cause Treg exhaustion, which might explain the lower abundance of neonatally-tagged Tregs in SLOs and NLTs. Additionally, both LPS- and poly I:C-challenged mice showed a long-lasting down-regulation of *Il7r* expression. Given the critical role of IL-7/IL-7R signaling for cell survival, it is reasonable to speculate that the lower abundance of neonatally-tagged Tregs is at least partially due to an impaired survival.

Recently, Rudensky et al. have generated mice harboring a reversible Foxp3^loxP-Thy-1.1-STOP-loxP-GFP^ reporter-null allele, which allowed them to restore Foxp3 expression in settings of established systemic inflammation by manipulating the time and duration of 4-hydroxytamoxifen treatment (34). Using this novel mouse model, they demonstrated that Tregs that developed 3 weeks after birth in a severely inflammatory environment could maintain a diverse TCR repertoire and confer long-term containment of autoimmune inflammation. In contrast to that, we observed a markedly reduced TCR diversity in neonatally-tagged Tregs generated under transient, TLR-agonist-mediated inflammatory conditions. One possible explanation for this discrepancy is that the long-term TCR diversity reduction might be restricted to inflammatory challenges occurring during a very narrow early-life time window since a shift of the inflammatory perturbation from day 1 to day 8 after birth already abrogated the long-term reduction of neonatally-tagged Tregs in SLOs and NLTs. It is also important to note that although both LPS and poly I:C are TLR agonists, the markedly reduced TCR diversity was observed only in LPS-and not poly I:C-treated mice. This might be due to a weaker perturbation strength elicited by the poly I:C dose applied in this study as we observed a more delayed and milder phenotype among neonatally-tagged Tregs in poly I:C- compared to LPS-treated mice. Furthermore, we cannot exclude the possibility that LPS might even act directly on the first wave of Tregs since it had been reported that Tregs express TLR4 (recognizing LPS) but not TLR3 (recognizing poly I:C) (44).

A limitation of the present study is that the long-term effect of neonatal inflammatory perturbations on the functionality of neonatally-tagged Tregs cannot be easily dissected by *in vitro* or *in vivo* studies due to their extremely small cell numbers. Generation of TCR transgenic mouse lines, similar to the strategy used in the visceral adipose tissue Treg study from the Mathis and Benoist group (28), might help to get a reasonable number of neonatally-tagged Tregs for subsequent functional studies. On the other hand, the biological consequence of the extraordinarily long-term reduction of neonatally-tagged Treg with lower *Foxp3* and *Il2ra* expression and diminished TCR diversity in both SLOs and NLTs is of great interest and awaits further investigation. Considering the crucial role of neonatally-generated Tregs in preventing autoimmunity and maintaining tolerance towards commensals, one could speculate that neonatally infected individuals have a higher risk to develop autoimmune diseases during adulthood.

Collectively, we reported different accumulation kinetics of neonatally-tagged Tregs in SLOs and NLTs under steady-state conditions and showed a decreased abundance of neonatally-tagged Treg with altered transcriptome and markedly diminished TCR diversity phenotype in both SLOs and NLTs upon neonatal inflammatory perturbations. Together, our data demonstrate that a single, transient encounter with a pathogen in neonates can have long-lasting consequences for the first wave of Tregs, which might affect immunological tolerance, prevention of autoimmunity, and other non-canonical functions of tissue-resident Tregs.

## Data availability statement

The datasets presented in this study can be found in online repositories. The names of the repository/repositories and accession number(s) can be found below: Gene Expression Omnibus (GEO), accession ID: GSE205466.

## Ethics statement

This study was reviewed and approved by Lower Saxony Committee on the Ethics of Animal Experiments and Lower Saxony State Office of Consumer Protection and Food Safety under the permit number 33.19-42502-04-17/2382.

## Author contributions

Conceptualization: JY, MZ, and JH. Methodology: MZ and JY. Formal analysis: JY, MZ, XC, YL, and MD. Project administration: SF. Writing - original draft: JY and MZ. Writing - review and editing: JY, MZ, MD, and JH. Funding acquisition: JY, MZ, and JH. Supervision: JH. All authors contributed to the article and approved the submitted version.

## Funding

This work was supported by the Helmholtz-Gemeinschaft (Future Theme “Aging and Metabolic Reprogramming”, ZT-0026), the Ministry for Science and Culture of Lower Saxony (research consortium COALITION), the Alexander von Humboldt Foundation through a post-doc fellowship to JY, the China Scholarship Council (CSC) through the State Scholarship Fund to MZ, and funded by the Deutsche Forschungsgemeinschaft (DFG, German Research Foundation) under Germany’s Excellence Strategy – EXC 2155 - project number 390874280.

## Acknowledgments

We thank Dr. Lothar Gröbe and Maria Höxter for cell sorting, the HZI Genome Analytics facility for sequencing, HZI Central Animal facility for support in handling animals, Friederike Kruse, Maria Ebel and Beate Pietzsch for technical assistance, and Yassin Elfaki, Joern Pezoldt, Lianxu Hao for scientific discussions.

## Conflict of interest

The authors declare that the research was conducted in the absence of any commercial or financial relationships that could be construed as a potential conflict of interest.

## Publisher’s note

All claims expressed in this article are solely those of the authors and do not necessarily represent those of their affiliated organizations, or those of the publisher, the editors and the reviewers. Any product that may be evaluated in this article, or claim that may be made by its manufacturer, is not guaranteed or endorsed by the publisher.
