# Supplementary material for: Inflammatory perturbations in early life long-lastingly shape the transcriptome and TCR repertoire of the first wave of regulatory T cells
Source: Front Immunol. 2022 Aug 31;13:991671. doi: 10.3389/fimmu.2022.991671 (PMC9471859; doi:10.3389/fimmu.2022.991671)
Supplement: Supplementary file 1 [file DataSheet_1.pdf]

Supplementary Material

Inflammatory perturbations in early life long-lastingly shape the transcriptome and TCR repertoire of the first wave of regulatory T cells

Juhao Yang, Mangge Zou, Xiaojing Chu, Stefan Floess, Yang Li, Michael Delacher, Jochen Huehn

Supplementary Figures

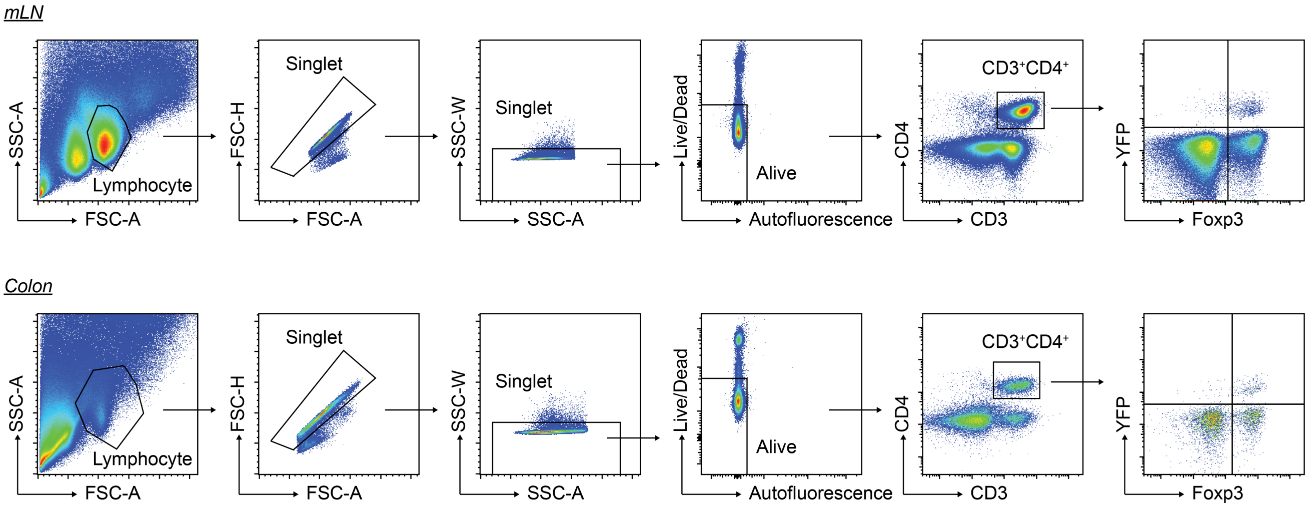

Supplementary Figure 1. General gating strategy. Gating strategy of neonatally-tagged Tregs in mLN and colon: Cells were first gated according to the forward (FSC) and side scatter (SSC) area parameters; doublet exclusion was performed using height (H) versus width (W) parameters of FSC and SSC accordingly, and Live/Dead discrimination was performed using LIVE/DEAD Fixable Blue Dead Cell Stain. After removal of cells with autofluorescence, $CD3^{+}CD4^{+}$ T cells were gated for determining Foxp3 and YFP expression.

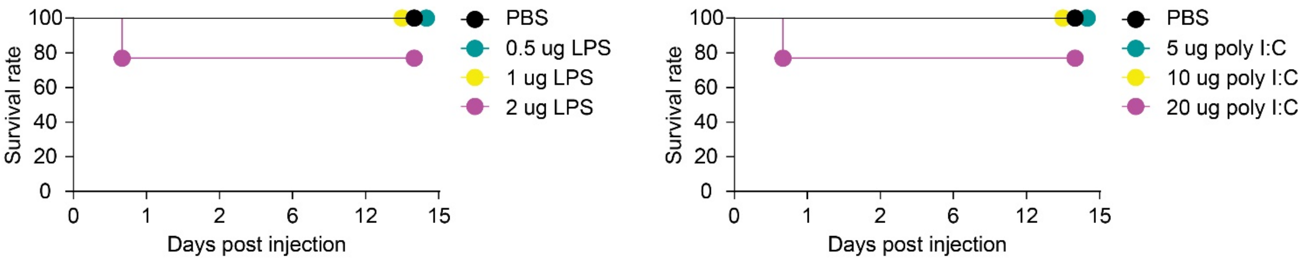

Supplementary Figure 2. Dose titration of LPS and poly I:C for establishing neonatal inflammatory perturbation models. Newborn Foxp3^{eGFPCreERT2}xROSA26$^{STOP\text{-}eYFP}$ mice were intraperitoneally injected with LPS, poly I:C or PBS as control, followed by repetitive intragastric injections of tamoxifen at days 2, 5 and 8 after birth. Graphs show survival rates upon treatment with indicated doses of LPS (left) and poly I:C (right). Data were pooled from 3 independent experiments (n = 5-6).

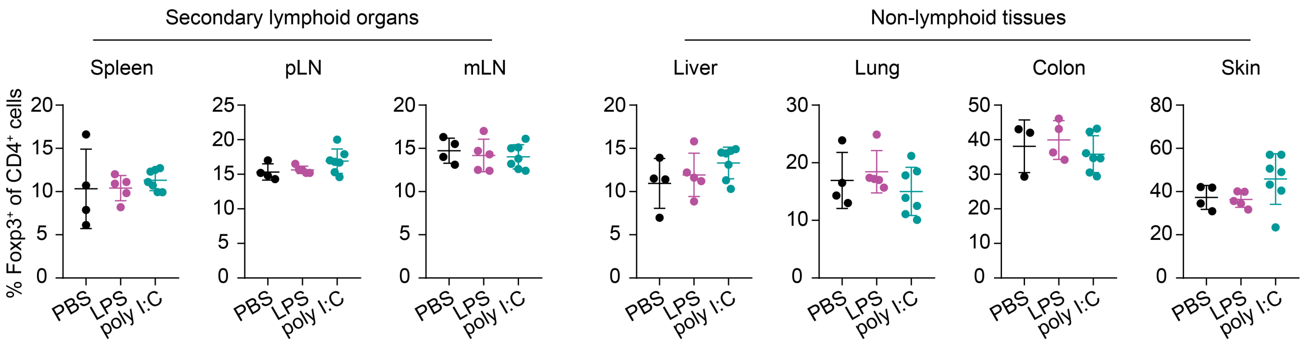

Supplementary Figure 3. No long-term impact on total Treg population upon later challenge. Foxp3^{eGFPCreERT2}xROSA26$^{STOP\text{-}eYFP}$ mice received repetitive intragastric tamoxifen injections at days 2, 5 and 8 after birth, directly followed by an intraperitoneal injection of LPS, poly I:C or PBS on day 8. Twelve weeks later, mice were sacrificed for immunophenotyping. Scatter dot plots summarize frequencies of Foxp3$^+$ cells among CD4$^+$ T cells in secondary lymphoid organs (left) and non-lymphoid tissues (right). Data in scatter dot plots are depicted as mean ± SD, each dot represents a single mouse, and data were pooled from 2 independent experiments (n = 4-7).

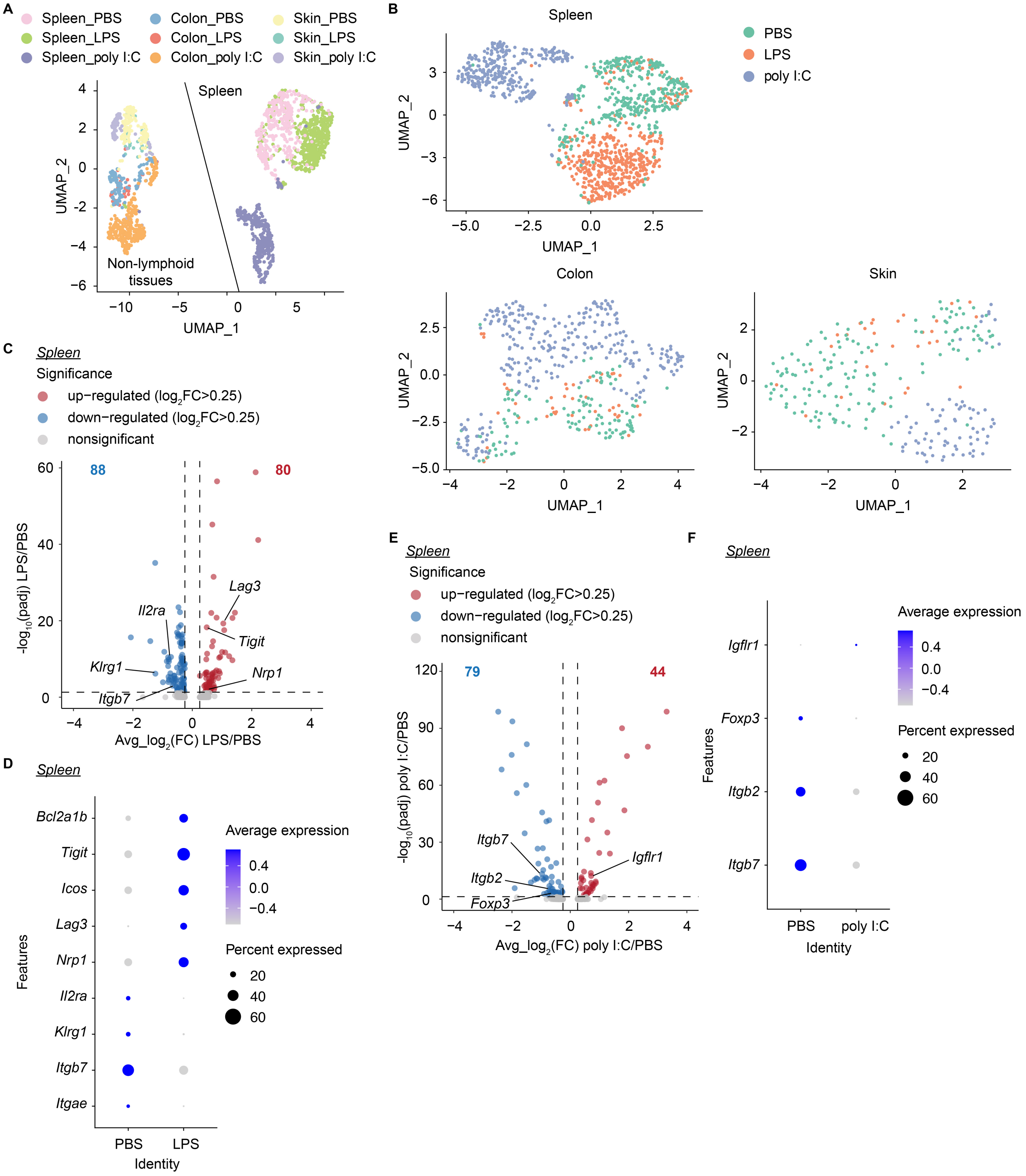

Supplementary Figure 4. Validation of long-lastingly altered transcriptomic profiles of neonatally-tagged Tregs upon neonatal challenge. Newborn Foxp3^{eGFPCreERT2}xROSA26$^{STOP\text{-}eYFP}$ mice were intraperitoneally injected with LPS, poly I:C or PBS as control, followed by repetitive intragastric injections of tamoxifen at days 2, 5 and 8 after birth. Twelve weeks later, YFP^{+} cells were FACS-sorted and subjected to combined scRNA/TCR-seq (the second batch of samples). **(A)** UMAP plot of merged YFP^{+} cells from spleen, colon, and skin of PBS-, LPS- and poly I:C-treated mice.

(B) UMAP plots of merged YFP$^+$ cells across organs of PBS-, LPS- and poly I:C-treated mice. **(C)** Volcano plot depicts avg_log$_2$(FC) vs. -log$_{10}$(padj) of identified DEGs between LPS and PBS conditions in the spleen. **(D)** Dot plot shows the expression of selected genes in splenic YFP$^+$ cells of PBS- and LPS-treated groups. **(E)** Volcano plot depicts avg_log$_2$(FC) vs. -log$_{10}$(padj) of identified DEGs between poly I:C and PBS conditions in spleen. **(F)** Dot plot shows the expression of selected genes in splenic YFP$^+$ cells of PBS- and poly I:C-treated groups. UMAP, uniform manifold approximation, and projection; FC, fold change; DEG, differentially expressed gene.

12 wk

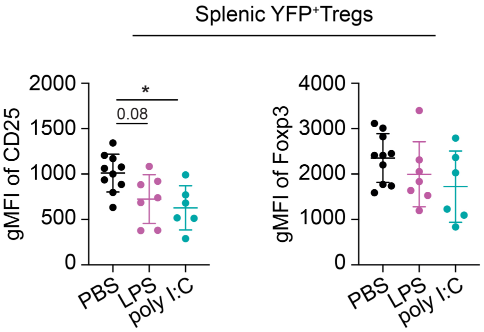

Supplementary Figure 5. Validation of the expression intensity of *Il2ra* and *Foxp3* at the protein level. Newborn Foxp3^{eGFPCreERT2}xROSA26$^{STOP\text{-}eYFP}$ mice were intraperitoneally injected with LPS, poly I:C or PBS as control, received repetitive intragastric injections of tamoxifen at days 2, 5 and 8 after birth, and were sacrificed 12 weeks post challenge for flow cytometric analysis. Scatter dot plots summarize the gMFI of CD25 and Foxp3 in splenic YFP^{+}Tregs. gMFI, geometric mean fluorescent intensity.

A

Treatment	Cells containing both TCRα and TCRβ chains	Total TCR types
PBS	272	149
LPS	230	55
poly I:C	207	135

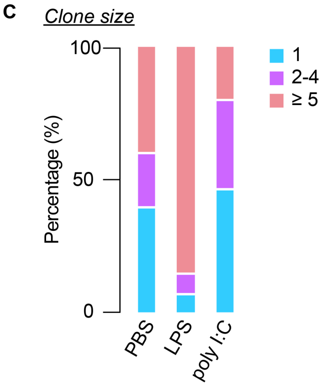

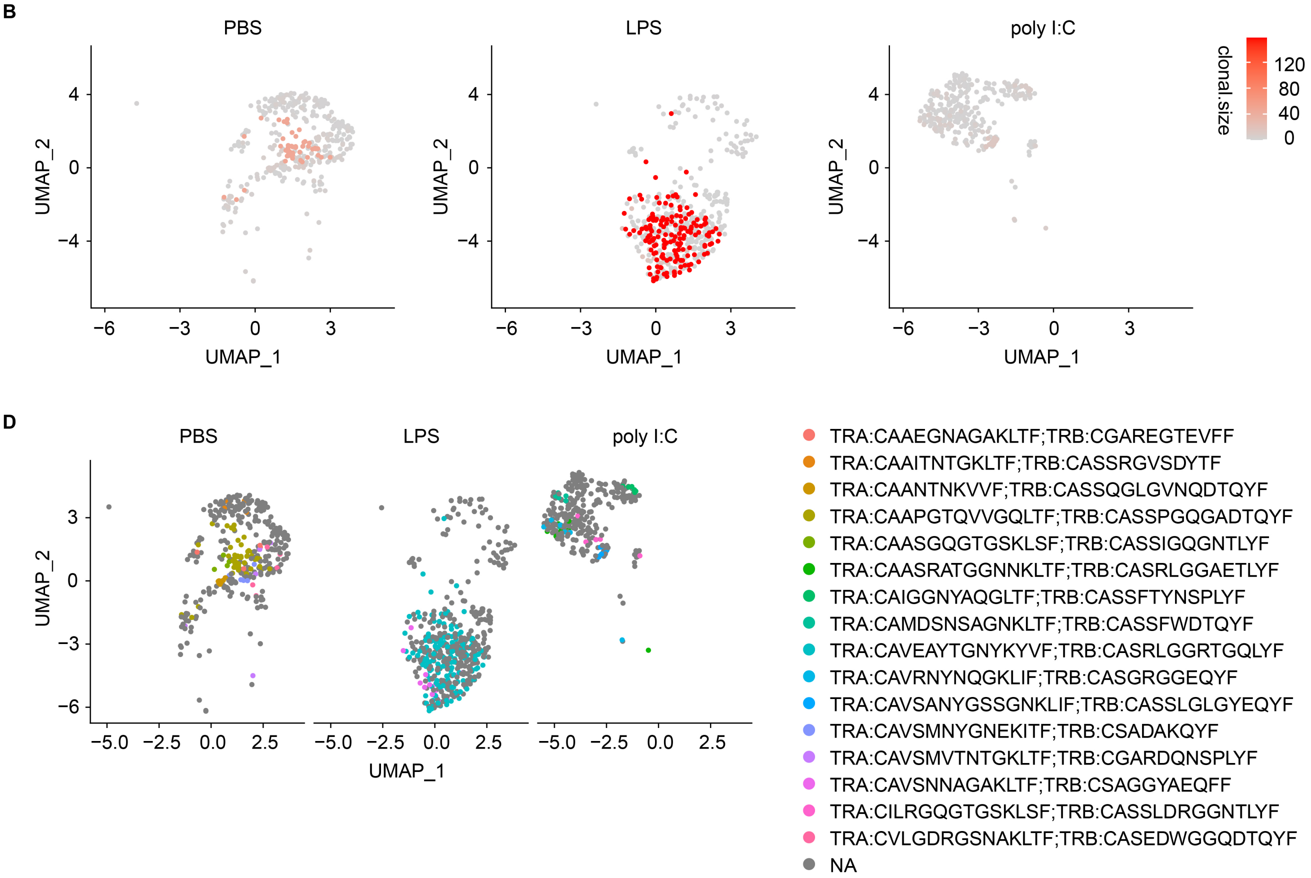

Supplementary Figure 6. Validation of diminished TCR diversity among neonatally-tagged Tregs 12 weeks post neonatal challenge. Newborn Foxp3^{eGFPCreERT2}xROSA26$^{STOP-eYFP}$ mice were intraperitoneally injected with LPS, poly I:C or PBS as control, followed by repetitive intragastric injections of tamoxifen at days 2, 5 and 8 after birth. Twelve weeks later, YFP$^+$ cells were FACS-sorted and subjected to combined scRNA/TCR-seq (the second batch of samples). **(A)** Table of total TCR clonotypes detected among splenic YFP$^+$ cells with paired TCRα and TCRβ chains detected in PBS-, LPS- and poly I:C-treated mice. **(B)** UMAP plots depict the clonal size in splenic YFP$^+$ cells. **(C)** Bar plot summarizes frequencies of cells with unique (n = 1) or clonal TCR (n = 2-4 and n ≥ 5). **(D)** Splitted UMAPs depict clonotypes detected in clonal YFP$^+$ cells (n ≥ 5). UMAP, uniform manifold approximation and projection.
